# Non-rigid registration of a 3D ultrasound and a MR image data set of the female pelvic floor using a biomechanical model

**DOI:** 10.1186/1475-925X-4-19

**Published:** 2005-03-18

**Authors:** Janko F Verhey, Josef Wisser, Simon K Warfield, Jan Rexilius, Ron Kikinis

**Affiliations:** 1 Department of Medical Informatics, University Hospital Goettingen, Germany; 2 Department of Obstetrics, University Hospital Zuerich, Switzerland; 3 Surgical Planning Laboratory, Department of Radiology, Brigham and Women's Hospital, Boston, USA; 4 MeVis – Center for Medical Diagnostic Systems and Visualization, Bremen, Germany

## Abstract

**Background:**

The visual combination of different modalities is essential for many medical imaging applications in the field of Computer-Assisted medical Diagnosis (CAD) to enhance the clinical information content. Clinically, incontinence is a diagnosis with high clinical prevalence and morbidity rate. The search for a method to identify risk patients and to control the success of operations is still a challenging task. The conjunction of magnetic resonance (MR) and 3D ultrasound (US) image data sets could lead to a new clinical visual representation of the morphology as we show with corresponding data sets of the female anal canal with this paper.

**Methods:**

We present a feasibility study for a non-rigid registration technique based on a biomechanical model for MR and US image data sets of the female anal canal as a base for a new innovative clinical visual representation.

**Results:**

It is shown in this case study that the internal and external sphincter region could be registered elastically and the registration partially corrects the compression induced by the ultrasound transducer, so the MR data set showing the native anatomy is used as a frame for the US data set showing the same region with higher resolution but distorted by the transducer

**Conclusion:**

The morphology is of special interest in the assessment of anal incontinence and the non-rigid registration of normal clinical MR and US image data sets is a new field of the adaptation of this method incorporating the advantages of both technologies.

## Background

In a recent study the advances of 3D sonographical imaging techniques to allow a sophisticated study of anal sphincter and levator ani muscle anatomy were described [1]. Today's common US examiniation techniques using a 7.5 MHz transducer allow a spatial resolution of up to 0.3 mm in each direction [2,3], whereas it is hard to obtain good quality MR images better than 1 mm in a single direction, when imaging the pelvis. Nevertheless, MR is a well established 3D data acquisition technique, which is used as gold standard to describe human anatomy in vivo.

It is a clinical necessity to enhance the information contained in imaging for diagnostic and also therapeutic purposes. In the past this led to new imaging techniques (visual representations) which use the information of at least two modalities in order to maximize the benefit for the clinician in diagnosis and treatment [4]. The registration of MR and US is of special interest because sonography is a diagnostic technique which is easy to handle, widely available, and furthermore economic [5]. To combine the best of the two worlds we will show that it is possible to match 3D MR and US for the assessment of female pelvic floor morphology.

Both introitus sonography and endoanal sonography focus on rectum and anal sphincter muscle morphology [6,7]. A recent publication by Williams et al. [8] shows a good correlation of endosonographic anatomy with endocoil MR. In contrast to this paper, our case shows the anatomy in a native physiologic state without stretching the tissue with a transrectal probe. No results have been published so far about the combination of introitus sonography and MRI using a standard bodyflex coil used for clinical purposes showing the anatomy in native status.

Because the organs in pelvic floor area are movable in position and size, consequently, new imaging techniques in this region should be based on non-rigid registration techniques (techniques considering tissue deformations) rather than on rigid registration techniques [9-11] in order to find the relationship of corresponding data set points. Actually, few cases of non-rigid MR and US image registration in the pelvic floor area are reported (e.g. for the treatment of prostate cancer by Mizowaki et al. [12]).

Non-rigid registration has become a fundamental method for medical image analysis during the past years [5,13-15,35] and is tested and approved with data from different anatomical regions [15-18]. An important issue in the registration process is the generation of deformation fields that reflect the transformation of an image in a realistic way with respect to the given anatomy [19,20]. Various physically based elastic models and algorithms have been recently described [21-24]. The aim of this paper is to apply an advanced algorithm previously approved for computer-assisted neurosurgery [25] and tested in corresponding head-neck data sets [26] now for registration purposes in the pelvic floor area, especially of the anal canal region to create a new visual representation.

Information derived from this type of image registration could lead towards new diagnostic (or even therapeutic) methods in the treatment of female pelvic floor dysfunctions using the MR data as a frame for high-resolution US data.

## Materials and methods

For our case report two corresponding 3D data sets (MR and US) of a women attending the outpatient clinics for diagnosis and treatment of urinary incontinence were taken with no specific clinical preference out of an ongoing study.

The 3D volume data set was acquired using Voluson 530 D, Kretztechnik, Zipf, Austria as it has been previously described [1]. The volume data set of the undistended anal sphincter and levator ani muscle was taken with a 7.5 MHz transvaginal probe (opening angle of 105° in tranversal and of 100° in longitudinal direction, isotropic resolution of 0.3 mm in each spatial direction) was placed at the posterior frenulum of labia minora.

The MR examination was carried out in sitting position with an 0.5T open configuration MR system, Signa SP GEMS, using a bodyflex surface coil for data acquisition. After a locator sequence, axial and sagittal T2-weighted fast-spin-echo sequences (TR 4000, TE 100, Matrix 256 × 256, slice thickness 7.2 mm, intersection gap 1.2 mm) were acquired and stored in DICOM format. The resolution in the matrix is 1.09 mm, whereby the MR data sets result to be non-isotropic in the three directions in space in contrast to the sonographical data sets having isotropic voxel size.

As a base system for both alignment and visualization we used the 3D-Slicer 2 software available free for non-profit organizations on both standard MS Windows platforms and Sun Solaris 5.8 Workstations [27-29]. The 3D Slicer software is designed for both diagnostical visualization and surgical planning, and it integrates several facets of image-guided medicine into a single environment: It provides capabilities for (I) automatic registration (aligning data sets), (II) semi-automatic segmentation, (III) generation of 3D surface models (for viewing the segmented structures), (IV) 3D visualization, and (V) quantitative analysis (measuring distances, angles, surface areas, and volumes) of various medical scans.

**Figure 1:**
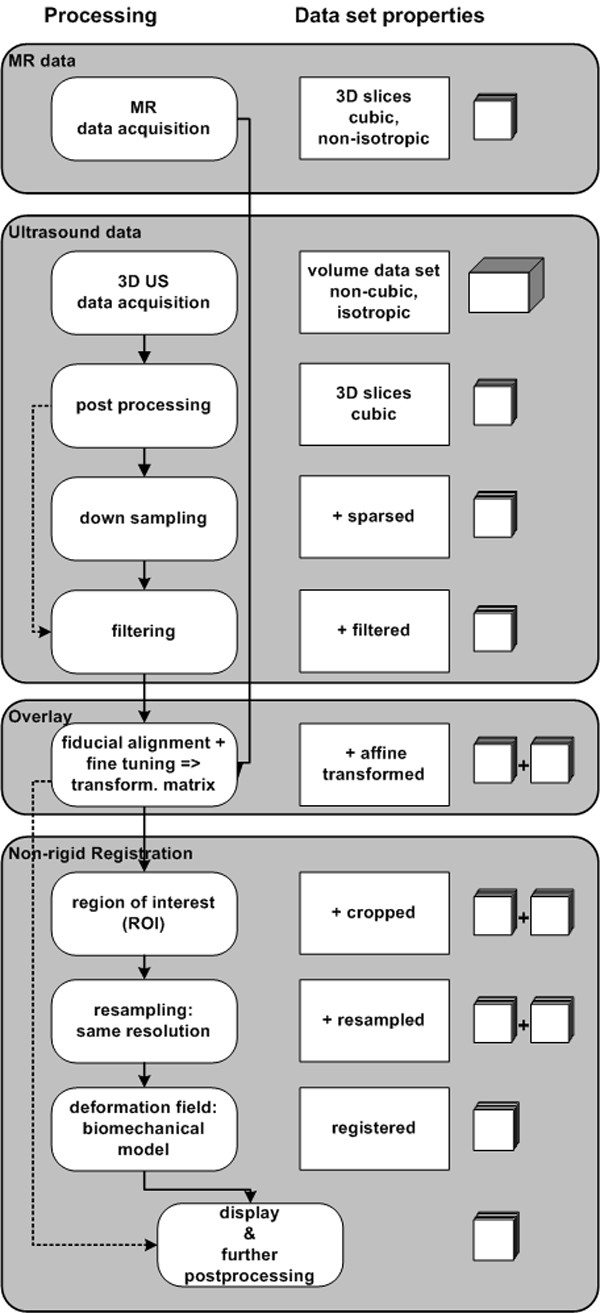
**Strategy to register**. Strategy to register MR and 3D ultrasound image data sets. Solid lines display the strategy shown in this paper. Dashed lines symbolize optional ways which were followed but which show no further relevant and new details. The symbols on the right refer to the format of the data sets: The series of squares stands for a series of parallel slices and the cube is for a volume block format.

**Figure 3:**
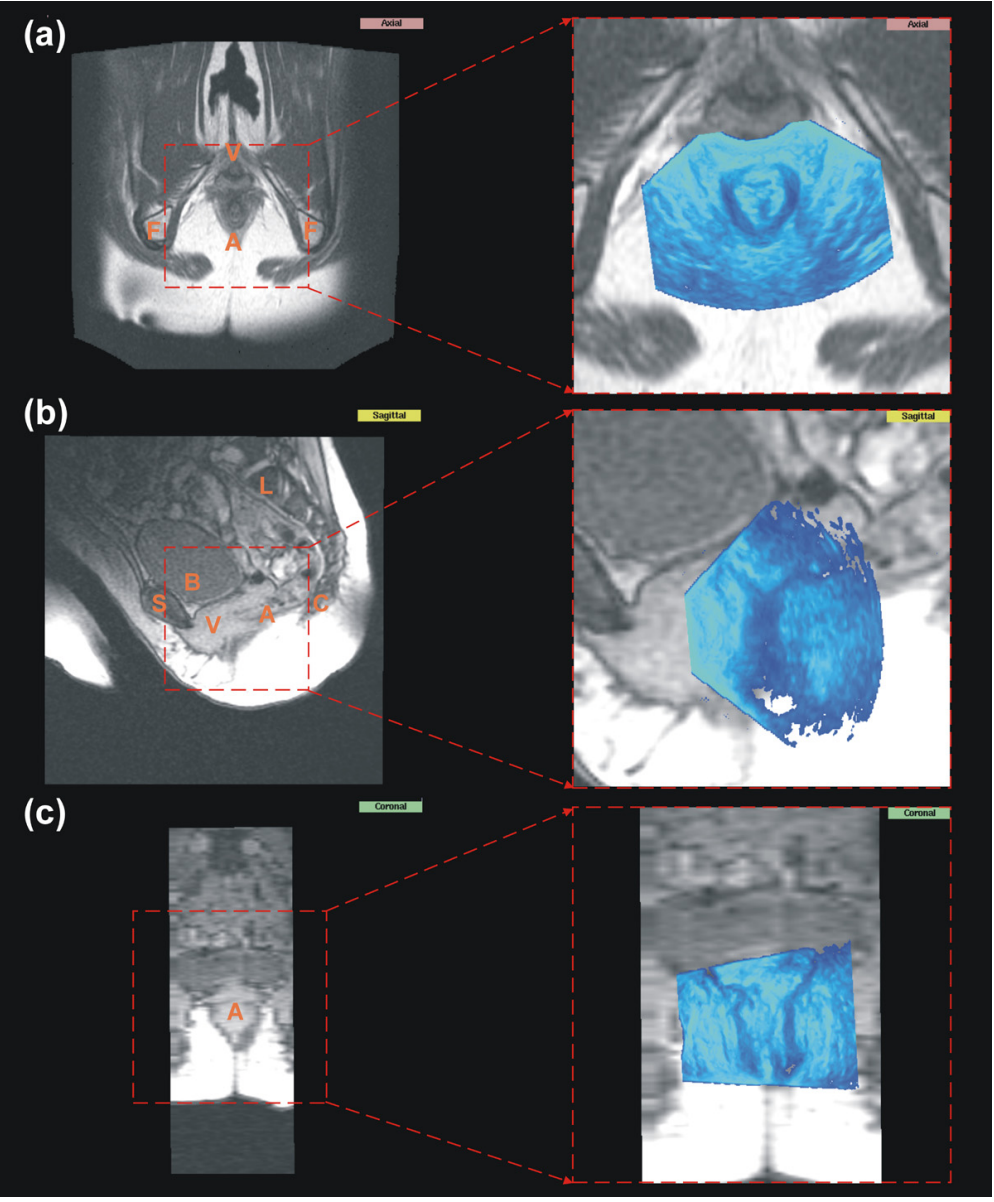
**MR data with aligned sonographical data**. Shown are the corresponding MR data set of the pelvic floor to the US data set from Fig. 2. (a) shows the axial plane from the axial data set; (b) and (c) show the sagittal respectively coronal plane from the corresponding sagittal data set. (c) is shown only for illustration purposes due to the poor resolution. On the right side the original MR data set is shown and on the left side a cutout with the corresponding initial alignment. The capital letters indicate anatomical structures: A: anal region; B: bladder; C: coccyx; F: ischial tuberosity; L: lumbar vertebrae; S: symphysis; V: vaginal region.

The processing followed the strategy as described in Fig. 1. After carrying out an edge enhancement in the 3D US data set using adaptive filter techniques [30,31] both datasets were initially aligned using the standard 3D-Slicer's fiducial alignment method by placing three landmarks as fiducials in two different axial slices of both axial MR and US data set. We chose the mucosa and points in the internal or the external sphincter muscle as anatomical landmarks for the rigid overlay assuming the MR images to be the gold standard for the muscle components [8]. As a result an affine transformation matrix was determinded for the overlay. Both data sets were cropped to the region of interest (Fig. 3) to minimize the calculation time. After resampling using a standard linear interpolation method both data sets have an isotropic resolution of 0.6 mm in each spatial direction. The worst registration errors of the correlation ratio are due to the MR resolution [32,33]. We carried out a visual assessment of the accuracy of the registration and assumed the error for each modality to be of the order of half a voxel size in our case [34]. The entire processing time for the process shown in Fig. 1 for this feasibility study was one hour not considering the data acquisition times for MR and US.

In this work we present the application of an algorithm for non-rigid registration described previously by two of the authors [19,20]. This algorithm was originally developed for MR techniques and we applied it for new anatomical region assuming that the edge enhancement algorithms are valid for US data sets, too. In order to obtain realistic deformations, we propose a physics-based elastic model. The method does not require segmentation and does not have the drawback that initial estimates of the deformation are only generated for the boundary of a considered structure. Instead, these estimates are calculated based on a template matching approach with a local similarity measure. Furthermore, we incorporated different models for elasticities into our algorithm. The discretization of the underlying equation is done by a finite element technique, which has become a popular method for medical imaging applications [24,25].

The registration process can be described as an optimization problem. Target of the optmization is the minimization of the deformation energy between two data sets, reference and template. The displacement field which describes the correlation of corresponding anatomical structures in both image data sets can be described using the theorema of minimal potential enegy E. With this in a volume Ω a deformation u needs to be determined which minimizes the following equation.



F means the external force causing the deformation u. σ is the stress which causes the (local) distortion ε. The relationship between σ and ε can be described using elastomechanical equation σ = Dε with D being an elasticity tensor. Due to the fact that in the dedicated anatomical region mostly muscle tissue is found, the tissue parameter in D used in the biomechanical model is assumed to be homogeneous (according to [20,22]and[25]). The minimization of the potential energy E then is the registration process devided in two basic steps. The method is described in detail in [20].

## Results

Leading structures in both of our data sets are the rectum and the anal canal with the mucosa, the circular as well as the conjoined longitudinal muscle layer of the anorectal junction and the levator ani muscle, especially the puborectalis muscle. These morphological structures can be identified clearly in US as well as in MR images – in the latter case with much less contrast than in the US data sets. In addition to this MR shows up the pelvic bones and skin surface facilitates spatial orientation.

**Figure 2:**
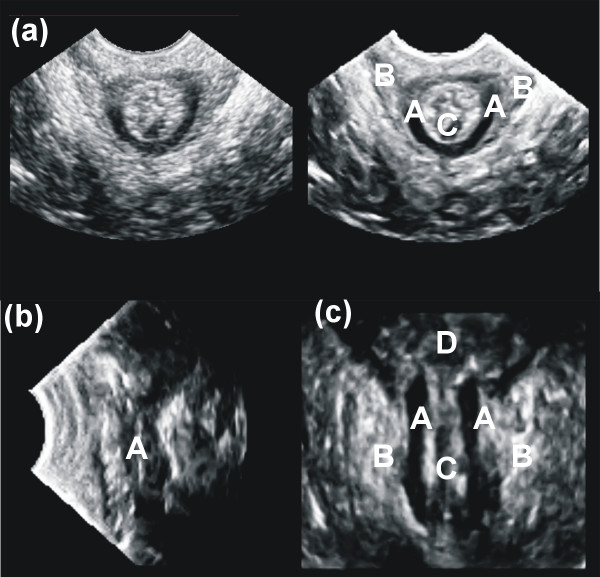
**Sonographical data**. Sonographical documentation method for examination of the pelvic floor in analogon to the usual used MR nomenclature [36, 37]. Shown is the filtered US data set: (a) axial, (left side: not filtered to show the enhancement induced by filtering) (b) sagittal and (c) coronal plane through the anal canal. A: the internal anal sphincter muscle; B: the external anal sphincter muscle (levator ani muscle); C: anal canal mucosa; D: rectum.

The application of adaptive filtering on US data set increased the signal to noise ratio significantly. As a consequence edges appear enhanced in the filtered images showing therefore the anatomical details much clearer. This is shown in Fig. 2. Both external and internal anal sphincter muscle as well as the anal mucosa can be identified in filtered images. Especially, the internal anal sphincter muscle appears clearly as a hypoechogenic region. The levator ani muscle has a V-form surrounding the external anal sphincter muscle best visible in the axial plane. It can be easily distinguished from the hyperechogenic tissue of the external anal sphincter.

Fig. 3 (left side) shows the MR data set analog to the US images in Fig. 2 in its different planes. In the right side of Fig. 3 the position and the size of the overlayed US data set and the spatial orientation in the MR data set is shown. Due to the higher resolution of the US data set about three times more data points are visible in the overlay region in comparision to the MR image significantly magnifying the overlay region. Fig. 3b shows the sagittal plane of the MR data set. Few structures are distinguishable clearly in the vaginal and anal region due to the poor resolution and contrast. Even filter techniques could not significantly increase the contrast in this region in sagittal scan and are not shown therefore.

**Figure 4:**
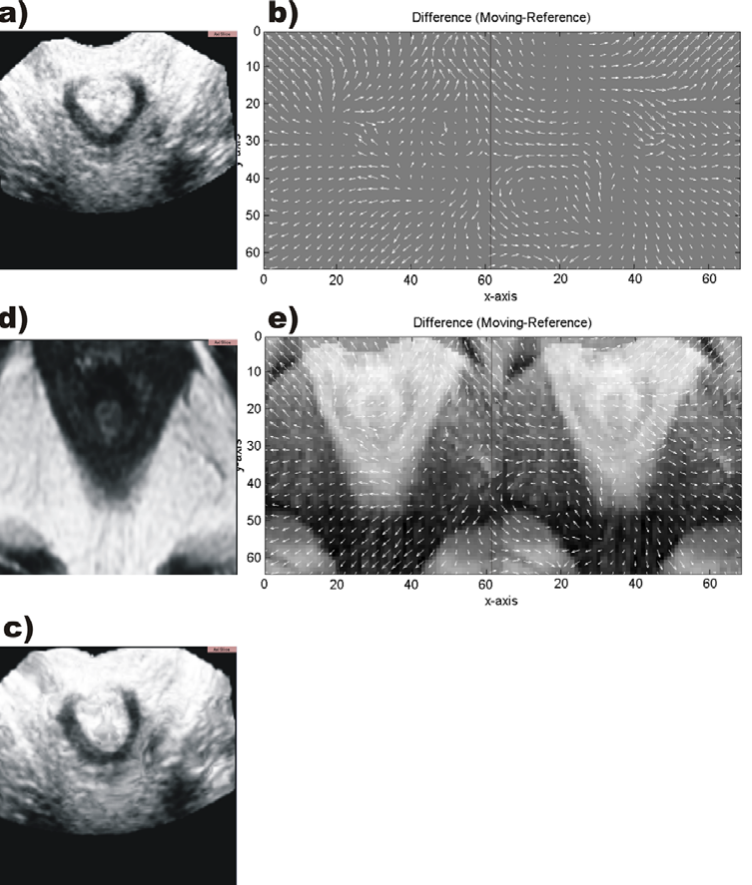
**Registration and displacement field**. Axial plane through non-rigid registration of the anal canal: Original data are shown in (a) and (d) – US and MR image, respectively. For simplification of visualization only the difference components in x-direction (b left) and in y-direction (b right) are shown. The difference in z-direction is ommited. (e) shows the difference image of (a) and (d) together with the difference components in x direction (e left) and in y direction (e right). (c) shows the registered image after the application of the displacement field.

In Fig. 4 the registration is explicitly shown for one axial plane. The top of each image indicates the anterior direction. MR is used as the template and US as the reference image. As a result the US data set is displayed in the coordinates of the MR due to the application of the deformation field (Fig. 4c). At the position of the transducer – best visible in the top of Fig. 4a – the displacement field shows major differences. The compression induced with the transducer head is partially corrected in the registered image Fig. 4c. The displacement field in z-direction perpendicular to the axial slice is ommited due to the poor resolution of the MR data set in this direction and due to the fact that in sagittal planes too few anatomical landmarks could be identified clearly.

**Figure 5:**
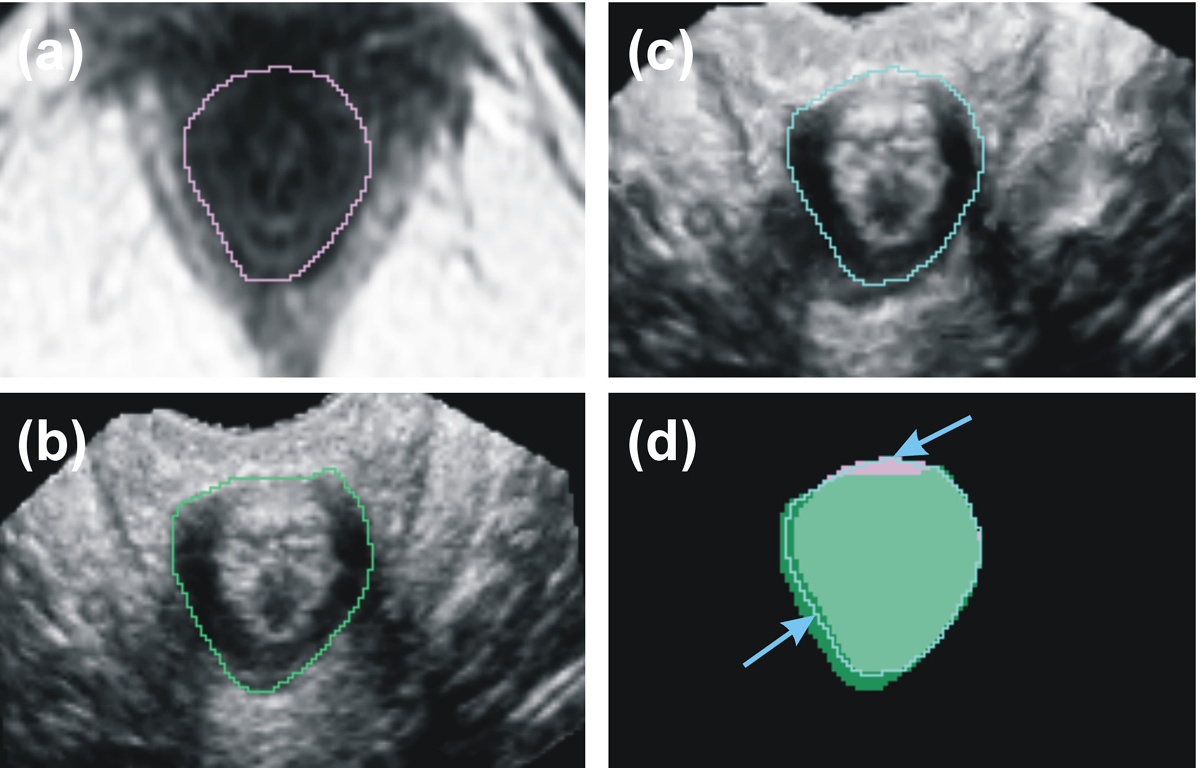
**Segmentation of the internal anal sphincter muscle**. Segmentation of the internal anal sphincter muscle. (a) shows the original axial MR slice with the contour of the internal anal sphincter muscle in pink color, (b) the corresponding US slice with the contour in green color and (c) the registered image with the contour in blue color respectively. (d) shows the areas in comparision. The arrows indicate where the contour of the internal anal sphincter muscle is modified with the algorithm. The registration partially corrects the compression induced by the transducer (the upper arrow shows the position of the transducer).

The non-rigid registration qualifies the visual assessment in axial plane very well. An estimation of accuracy in both sagittal and coronal plane could be quantified not better than half a voxel size. In Fig. 5 the segmentation of the internal anal sphincter muscle is explicitly shown in one axial plane. Analogous to Fig. 4 the top of each image indicates the anterior direction. It is shown for the MR (Fig. 5a), the US (Fig. 5b) and the registered image (Fig. 5c) respectively. The deformation induced by the ultrasound probe placed in anterior position is corrected (Fig. 5d).

## Discussion

MR leads to rigid non-deformed data sets in contrast to most sonographical data acquisition techniques. The better structural contrast of the MR data sets allows a better spacial orientation but the quality and resolution especially for soft tissue is higher with 3D US techniques.

Due to several factors such as the lack of image structure, the poor signal to noise ratio of the MR data set, the intensity artifacts, the computational complexity and the restricted time frame it is not feasible to quantify the deformation occurring between each voxel of the corresponding data sets directly. Consequently, we chose a physics-based non-rigid registration algorithm to estimate a deformation field only at sparse locations which have to be interpolated throughout the image. Models of this type have become popular for non-rigid registration because they are fast and have the potential to constrain the underlying deformation in a plausible manner.

Included in our method is an edge enhancement for the US data set using adaptive filtering. Our sophisticated 3D filter technique requires an isotropic or at least a nearly isotropic resolution of the volume image data sets which is the case for our US data set. Furthermore, most registration methods require to have similar resolution of both images, similar regions with the same image size and only local deformations on a short range scale. Therefore, nevertheless, preprocessing steps were needed. Corresponding clinical data sets of two different modalities usually fail fulfilling those preconditions completely – so do ours.

For initial alignment purposes it helps to localize the anatomy. This is demonstrated in Fig. 2. The difference of resolution in MR's axial direction in comparison to the resolution of ultrasound is huge. Due to this, a misplacement of ultrasound's axial slices in relationship with MR axial slices was of no initial relevance and our error in misalignment assumed to be in the region of half a voxel size in each spatial direction.

Fig. 3 shows the poor resolution and contrast in the vaginal and anal region of the MR data sets. Few structures are distinguishable clearly in this region. Even filter techniques could not significantly increase the contrast in this region in sagittal scan and are not shown therefore. This leads to the wish to enhance the clinical information in this region using registration techniques as presented with this paper.

MR and US show minor contour differences for all the anatomical structures. The reason for this misplacement is based on the two completely different data acquisition techniques. Even if they were very carefully carried out the prevention of any distortion is impossible and leads to the following effects: 1. The coupling of the transducer to the tissue induces a tissue deformation in any case. 2. The position of the patient varies without placing any external markers if the data acquisition takes place at different places and different times. 3. Different acquisition times induce e.g. different filling levels of the organs with different contours as a result.

The registration results shown in Fig. 4 and Fig. 5 demonstrate clearly the feasibility of the non-rigid registration method for the correspondent 3D data sets of the anal canal. The native MR data set is used as a frame for the US data set – which shows the sphincter structures more clearly than the MR.

The levator ani muscle cannot be registered with accuracy because the identification in the US data set due to the poor contrast needs an experienced clinician and results impossible for an automated algorithm. The visual representation is limited to slightly distorted tissue structures as proved with this data and shown before in previous attempts in corresponding head-neck data sets. But regions with high contrast and slight deformations can be registered using our method. For further studies MR data sets with higher resolution MR and isotropic voxel size would be desirable.

## Conclusion

The present case report shows the feasibility of the visual representation presented for normal clinical data of the anal canal. The registration partially corrects the compression induced by the transducer, so the MR data set showing the native anatomy is used as a frame for the US data set showing the same region with higher resolution but distorted by the transducer. As a consequence the clinical information for diagnostic purposes is enhanced for resolution (Fig. 3) and for position (Fig. 5).

Obviously, these findings need to be validated with more cases in a future prospective study. As previously emphazised the MR images were assumed to be the gold standard for the contours. Using the non-rigid registration technique described in this paper and developing a more sophisticated data acquisition and registering technique this might be changed in the future and the application of 3D ultrasound (US) has a high potential in the innovative development of future low-cost applications.

## Authors' contributions

JFV developed the registration strategy and did the technical part implementing the visual representation method described here. JW did the data acquisition and the medical part. SKW and JR developed the used linear elastic model and helped to modify and adapt it to the present data. RK provided the computing infrastructure and consulted the development process of the method. All authors read and approved the final manuscript.
